# Optimizing Broiler Performance and Intestinal Morphology and Increasing Nutritional Availability via Microbial Muramidase Supplementation

**DOI:** 10.3390/ani16071123

**Published:** 2026-04-07

**Authors:** Akram El Kadi, Radmila Marković, Dejan Perić, Sladjan Nešić, Nataša Glamočlija, Aurélia A. Séon Simon, Dragan Šefer

**Affiliations:** 1 Faculty of Veterinary Medicine, University of Belgrade, Bulevar Oslobodjenja 18, 11000 Belgrade, Serbia; radmilam@vet.bg.ac.rs (R.M.); dperic@vet.bg.ac.rs (D.P.); sladjan@vet.bg.ac.rs (S.N.); glamonata@gmail.com (N.G.); dsefer@vet.bg.ac.rs (D.Š.); 2 Research Center for Animal Nutrition & Health—DSM Nutritional Products France, 1 Boulevard d’Alsace, 68128 Village Neuf, France; aurelia.seon@dsm-firmenich.com

**Keywords:** muramidase, broiler performance, intestinal morphology, blood carotenoids, gastrointestinal functionality

## Abstract

An experiment was conducted in a commercial broiler farm to evaluate birds’ growth performance, intestinal morphology (IM), and total blood carotenoid levels when adding different inclusion rates of microbial muramidase (MUR) to broilers’ feed formula. A total of 336-day-old broilers from Ross 308 were assigned to three groups and were fed from day 1 to day 42. Treatment 1 (T1), the control treatment, was a basal corn soybean meal diet that followed Aviagen nutrient specifications; Treatment 2 (T2) was the control treatment + 350 g MUR per ton of feed; Treatment 3 (T3) was the control treatment + 450 g MUR per ton of feed. Body weight gain (BWG), feed intake (FI), the feed conversion ratio (FCR), carotenoid levels (CLs), and IM from samples of the duodenum, jejunum, and ileum were evaluated on day 42. MUR significantly outperformed the control diet in terms of body weight (BW), resulting in BWs of 2513 g (*p* = 0.005) and 2463 g with T3 and T2, respectively, versus 2377 g with T1. Daily weight gain (DWG) was 58.77 g and 57.58 g in T3 and T2 (*p* = 0.0004), respectively, versus 55.54 g in T1. The Daily Feed Intake (DFI) on day 42 was higher in T3 compared to T1 (98.90 g *p* < 0.0001 versus 97.72 g). The morphology results show higher effects in the duodenum with T3 versus T2 and T1. In addition, the results were significantly higher in the jejunum when using T2 and T3 compared to T1. Goblet cells were not influenced by MUR use in the diets. The broilers’ blood carotenoid levels were significantly higher in T3 (1.75 mg/L *p* = 0.0026) compared to T1 (1.02 mg/L). These results shed light on broilers’ performance, intestinal health, and nutrient availability when using MUR at different inclusion rates in broiler feed for broilers raised under near-commercial conditions.

## Simple Summary

The global poultry industry is facing a new and demanding challenge following the restriction on the use of production enhancers in animal feeds. This global legislative framework states that poultry producers must promote alternative feed additives to enhance bird performance levels and gut function and improve digestibility or nutritional availability. This study indicates that a new category of feed enzyme, muramidase, which targets and hydrolyzes peptidoglycans in bacterial cell walls, improves broiler performance in a dose-dependent manner at a near-commercial-farm level.

## 1. Introduction

The poultry production industry is currently undergoing a remarkable period of transformation. There is much discussion worldwide on phasing out antibiotics from animal production mainly due to concerns regarding antibiotic resistance and possible implications for animal and human health. As a result, the importance of maintaining good gut health in broilers is attracting more attention, as is the necessity of feeding not just the animal but also its gut microbiome [1]. Broiler performance, health, and welfare are determinants highly influenced by broiler gastrointestinal health [2]. Nutrient availability and the use of feed additives to improve gastrointestinal functionality are essential factors contributing to such performance factors [3]. Numerous studies have shown the positive effect on poultry performance of using dietary enzymes [4,5].

To further understand this, it is important to delve into the details of the gastrointestinal tract (GIT). Within the GIT are peptidoglycans (PGNs), fine structures forming the cell wall of bacteria that hold the bacterial content. The cell wall of bacteria is in a frequent recycling process during multiplication to recover released constituents by active transport [6,7,8]. As high amounts of PGNs are released from dead bacteria, they are accumulated along the epithelial surface of the enterocytes. These PGNs negatively impact the gut immune effectors and impair the digestion and absorption of nutrients [9]. A study evaluated the use of a microbial muramidase as a feed supplement to improve gastrointestinal functionality and obtain higher performance in broiler birds [10].

Muramidase is an enzyme used as a feed additive to cleave the β-1,4-glycosidic linkage between N-acetylmuramic acid and N-acetyl glucosamine in the PGN backbone of bacterial cell walls. This enzyme does not target a substrate present in the feed, but rather a substrate already present in the GIT, namely PGNs, which are degraded by lysozymes or N-acetyl muramidases, referred to in this study as Balancius^®^ [11].

Others have reported muramidases as lysozymes because they are also known as PGN N-acetylmuramoylhydrolase [12].

Studies on lysozymes date back to the early 1920s. In 1922, Alexander Fleming isolated the chicken muramidase from the hen albumen [13]. Recent studies demonstrate the potential benefits of supplementing muramidase as feed additives to rabbits [14], broilers, turkey [15], and pigs [16,17]. Muramidases are found abundantly in different animal secretions, plants, and microorganisms and are reported to modulate inflammatory responses in the GIT of different species [18].

Muramidase is a natural enzyme, which makes it safe for use in animal feed; enzymatic exposure of MUR in the animal GIT does not occur [19]. In addition, studies have confirmed muramidases enzymes to be abundant, showcasing one-layer eggs that contain around 100 mg of natural muramidase [20,21]. Toxicology studies have reported the safe use of the selected muramidases produced by Trichoderma reesei DSM 32338 (Balancius^®^). This Balancius^®^ Product is safe for use as a feed additive at the recommended level of up to 65,000 Lipase Substrate Units (Fluorescence-based) LSU(F) per kilogram of feed [22]. One LSU(F) unit is defined as the amount of enzymes that increases the fluorescence by 12.5 µg/mL fluorescein-labeled PGNs (purified from Micrococcus luteus and supplied by Novozymes A/S), a value that corresponds to the fluorescence of 0.077 mM fluorescein isothiocyanate per minute at pH 7.5 and 30 °C [19,22]. In addition, this muramidase has no antimicrobial activity, differentiating it from commercial lysozyme isolated from hen egg whites or that endogenously present in animal secretions [3,19].

This study aimed to investigate the effect of using different inclusion levels of muramidase on the performance of broilers raised in mini floor pens—intended to reproduce near-commercial conditions—by assessing the BW, FI, FCR, intestinal histomorphology, and blood carotenoid concentration of 42-day-old broilers.

## 2. Materials and Methods

### 2.1. Ethical Statement

The experimental design and protocol were approved by the Veterinary Directorate of the Serbian Ministry of Agriculture, Forestry and Water Management, and the Ethics Committee of the Faculty of Veterinary Medicine, University of Belgrade (Resolution number: 01-511-2/2020/07/23).

### 2.2. Broilers, Housing, Experimental Design, and Diets

This study was conducted in a single environmentally controlled house, in which all three treatment groups were housed together under identical management conditions. This required automatic temperature and humidity control, uniform lighting, and standardized feeding and drinking systems. Treatments were randomly assigned to pen positions to account for potential location effects. In addition, environmental parameters including temperature, humidity, and lighting were continuously monitored and maintained within recommended ranges throughout the 42-day experimental period, minimizing the likelihood of a significant pen location effect on the outcomes measured. At the end of the experiment (day 42), all the birds were weighed after an 8 h fasting period. Eight birds from each treatment were sent to the slaughter house, hung, and bled.

A total of 336 one-day-old Ross 308 broilers of both sexes were housed on arrival in a shed measuring 6 by 4 m and divided into three compartments of 8 m squared each. The experiment comprised 3 groups, each of which had 112 broilers divided into 8 pens, with 14 birds per meter squared and an average chick weight of 44 g each. The pens were distributed randomly in the shed and were divided with partitions to separate the replicates. The pens contained wood shavings with a maximum thickness of 3 cm and nipple drinkers (14 birds per nipple), as well as round drinkers and round feeders. The lighting system had a light intensity of 30 Lux, and the house was equipped with automatic temperature/humidity control throughout the period.

The farm management and experimental diets followed the recommendations of Ross 308 Aviagen. In this trial, broilers were collected from a commercial hatchery with same-aged parents and were not identified by sex at any time, so an approximately equal ratio of males and females is assumed for the distribution in the experimental groups.

Feed was administered as follows: a control diet Treatment 1 (T1), which consists of a plant-based diet of corn and soy bean meal; Treatment 2 (T2), the control diet plus muramidase at 35,000 LSU (F)/kg or 350 g/ton of feed; Treatment 3 (T3), the control diet plus muramidase at 45,000 LSU (F)/kg or 450 g/ton of feed. The muramidase was added as Balancius^®^ in the formula premix without changing the nutrient values of the three treatments starting on day 1 of the experiment (Table 1). Apo-ester, used as a biomarker of the intestinal absorptive capacity, was also added into the premix in the three treatments at 4 ppm (40 mg/kg) of Carophyll^®^ Yellow 10%, DSM Nutritional Products, Inc., Kaiseraugst, Switzerland. The carotenoid concentration was analyzed in the blood at the end of the study. The birds were fed a mash diet from the beginning until the end of the experiment: starter feed from day 1 to day 14, grower feed from day 15 to day 28, and finisher feed from day 29 to slaughtering on day 42.

Feed and water were provided ad libitum with no antibiotic supplementation during the experimental period in either. A recommended vaccine program was implemented against Newcastle disease on days 4 and 14 and against Gumboro disease on days 7 and 22.

The birds were inspected daily by a poultry veterinarian to assess their general health, to maintain ideal temperature, humidity, and lighting conditions, and to adhere to the recommended standards. The brooding temperature was set at 33 °C for the first week and reduced gradually by 2 °C weekly, reaching 22 °C at the end of the cycle. The humidity varied between 60% and 65%. The weight of each broiler was registered on their arrival day, on day 21, and finally on day 42. The lighting program for the first 7 days of age provided for a long day length of 23 h of light and 1 h of dark. From day 8 onwards, the broilers were subject to 6 h of darkness to ensure good feed intake and an actual amount of darkness to follow local legislation.

### 2.3. Muramidase

The muramidase was added as Balancius^®^ (DSM Nutritional Products, Kaiseraugst, Switzerland), produced by submerged fermentation of Trichoderma reesei microorganisms, with a muramidase activity of 100,000 LSU (F)/g, and was applied according to the manufacturer’s recommendations.

### 2.4. Histological Analysis

After slaughter, organ evisceration, and detailed macroscopic examinations, intestinal sections were sampled for histological and morphometric examinations. Intestinal tissue samples of the duodenum, jejunum, and ileum were taken from each group of chickens immediately after slaughter. Samples measuring 3–5 cm in size were fixed in 10% neutral formalin for 48 to 72 h. Formalin was injected into the lumen of the ligated intestine for better fixation and prevention of blinding and deformation of the intestinal wall. After fixation, the samples were routinely processed in an automatic tissue processor (Leica TP1020) Kedee (Jinhua, China) for dehydration through a series of alcohols, followed by illumination in xylene and impregnation with paraffin, after which they were embedded in paraffin blocks and cut into sections 3–5 µm thick. The sections obtained this way were stained with hematoxylin–eosin (HE) and Periodic acid–Schiff (PAS) staining.

### 2.5. Histomorphometry Analysis

An Olympus BX51 optical microscope from (Olympus, Tokyo, Japan), with a Color View III digital camera (Olympus) and Olympus Cell B image analysis software, was used for morphometric analysis (Serial Number A50053015D4AE). A Leica RM 2235 microtome (2006, Leica, Berlin, Germany), Leica EG 1120 paraffinator (1997, Germany), and Kedee KD-TS3D automatic tissue processor (2016, China) were used to prepare the tissue sections.

The height of the intestinal villi was measured on each slide on ten randomly selected, correctly oriented intestinal villi at 10x objective magnification. The height of the intestinal villi was measured in micrometers as the distance from the tip to the base of the intestinal villi.

The number of goblet cells per 100 enterocytes was measured using the Touch Count option by counting the goblet cells in the PAS-stained preparations and then the nuclei of the enterocytes in their surroundings.

### 2.6. Carotenoid Measurements

After raising the birds for 42 days, 1 bird was selected per pen (8 samples per treatment) to determine the total carotenoid concentration in their blood sample, which was used as an indicator of intestinal integrity and nutrient absorption. The carotenoid concentration was measured using an iCheck Carotene device with iEx Carotene reagent vials (BioAnalytGmbH, Teltow, Germany). In short, 0.4 mL of blood was injected into an iEx vial, mixed for 10 s, and left to rest for 5 min to extract the carotenoids from the samples.

### 2.7. Statistical Analysis

The results were statistically analyzed using the software GraphPad Prism version 6.00 for Windows (GraphPad Software, San Diego, CA, USA, www.graphpad.com (accessed on 15 May 2024)). All variables were expressed as mean and standard deviation (sd). One-way ANOVA with Tukey’s post hoc test was performed to assess significant differences among experimental groups. Statistical significance was considered at *p* < 0.05.

## 3. Results

### 3.1. Growth Performance of Broilers

The one-day-old broilers were selected at the beginning of the experiment to have a mean body weight (BW) of approximately 44 g. The effects of dietary muramidase supplementation on broiler growth performance are summarized in Table 2. By day 21, birds fed 350 g/ton muramidase (T2) had significantly higher BW (934 ± 92.39 g) compared to the control group (T1; 901.6 ± 80.04 g), while the 450 g/ton group (T3; 909.7 ± 87.29 g) was intermediate (*p* = 0.0152). On day 42, BW was significantly greater in the T2 (2463 ± 306.4 g) and T3 (2513 ± 214.5 g) groups compared to the T1 group (2377 ± 247.9 g; *p* = 0.0005).

Daily weight gain (DWG) from day 1 to day 21 was significantly higher with T2 (42.37 ± 4.3 g/bird) than with T1 (40.83 ± 3.71 g/bird), while T3 (41.21 ± 4.03 g/bird) was intermediate (*p* = 0.0119). From day 22 to day 42, T3 birds exhibited the highest DWG (76.33 ± 6.11 g/bird), significantly greater than both T1 (70.25 ± 8.07 g/bird) and T2 (72.79 ± 10.38 g/bird; *p* < 0.0001). Over the entire period (1–42 days), DWG was significantly improved in T2 (57.58 ± 7.24 g/bird) and T3 birds (58.77 ± 5.04 g/bird) compared to T1 birds (55.54 ± 5.85 g/bird; *p* = 0.0004).

Daily Feed Intake (DFI) from day 1 to day 21 was significantly lower in T2 (56.89 ± 0.334 g/bird) and T3 birds (57.56 ± 0.163 g/bird) compared to T1 birds (58.99 ± 0.763 g/bird; *p* < 0.0001). Conversely, from day 22 to day 42, DFI increased with muramidase, with T3 birds consuming significantly more (150.65 ± 0.984 g/bird) than T2 (148.62 ± 1.003 g/bird) and T1 birds (146.74 ± 0.339 g/bird; *p* < 0.0001). Over the entire period, T3 birds had the highest DFI (98.9 ± 0.12 g/bird), significantly greater than with T1 and T2 (*p* < 0.0001). The feed conversion ratio (FCR) did not differ significantly among treatments during any of the periods evaluated (*p* > 0.05).

### 3.2. Intestinal Morphology of Broilers

Details of the intestinal morphology of the broilers are displayed in Table 3. Specifically, the villus height, crypt depth, villus height/crypt depth ratio, villus width, and goblet cell density were measured in the duodenum, jejunum, and ileum.

In the duodenum, the villus height was higher in the T3 broilers (*p* < 0.0001). A higher villus width was also found in the T3 group compared to the T2 and T1 groups (*p* < 0.05). Also, the jejunums of the T2 and T3 broilers showed higher villus heights, villus/crypt ratios, and villus widths when compared to those of the T1 group (*p* < 0.05). Ileum histopathology was more notable in the T2 and T3 broilers than in the T1 broilers (*p* < 0.05).

The villus width in the duodenum, jejunum, and ileum in the T3 group was higher (*p* < 0.05) than that in the T2 and T1 groups. The goblet cell density in the three morphological sections was not significantly different between the T2 and T3 broilers. The goblet cell density in the ileum was higher (*p* < 0.05) among the broilers fed 0 g/ton of Balancius^®^.

### 3.3. Blood Carotenoids

The carotenoids that were used as a biomarker in the three feeds can be measured easily and quickly on floor-reared broilers. As shown in Table 4, the results of T3 showed higher levels compared to T2 and T1 (*p* < 0.05).

Broilers fed diets supplemented with T3 showed a higher mean (1.75 ± 0.67 mg/L; *p* < 0.0026) concentration of carotenoids in the blood on day 42 compared to T1 and T2 (1.02 ± 0.21 mg/L and 1.08 ± 0.31 mg/L).

## 4. Discussion

In this study, adding muramidase to broilers’ feed diets improved their growth performance. Treatments with MUR as a feed additive show a significant increase in BW, DWG, and DFI. These results are comparable to those of other studies reporting positive responses in growth performance while using MUR in broiler diets [23]. The beneficial effects of MUR use on broiler performance have been shown to support our results on growth performance [24,25]. However, our results are not compatible with those of a study stating that MUR use had no positive effect on growth performance or showed any significant effect on the FCR on day 21 or day 42 [1,2]. The FCRs we obtained were 1.78, 1.72, and 1.69, respectively, in T1, T2, and T3, which are also incompatible with the findings of some studies [2,19,25,26]. In these instances, the FCR obtained with broilers fed MUR at 350 g/ton was significantly lower than for the control diet. Some studies have shown improved performance in broilers fed a higher dose of MUR at 450 g/ton, but they found no change in the FCR compared to 350 g/ton of MUR [19,25,26]. In the present study, the observed reduction in Daily Feed Intake (DFI) during the early phase (days 1–21) in muramidase-supplemented groups (T2 and T3) suggests an initial improvement in nutrient utilization efficiency, consistent with the known mechanism of muramidase in degrading bacterial peptidoglycan within the gut, thereby reducing the metabolic cost of immune activation and redirecting energy partitioning toward productive growth [15].

The variability in the response in bird feed intake, growth performance, and feed conversion ratio shown in different studies using MUR may be influenced by the diet formulation, enzyme dosage, bird age and breed, management program, environment, and species. Given this diversity, in our study at the near-commercial-farm level, we can speculate that the mode of action or the response could also be different.

In addition, significant growth performance was observed on day 21 in response to dietary MUR, and it continued to increase as the birds aged to day 42. This suggests that the beneficial effect of MUR is related to the changes in broiler gut microbiota as the bird grows. In a similar study, the numbers of microbes reached 1011/g of cecal digesta and 109/g of ileal digesta during the first three days post hatch, and these microbes remained relatively stable for the following 34 days [27]. As a bird grows, it consumes higher amounts of feed, the size of its GIT increases, and the total number of microbes in the GIT will increase proportionally. One author stated that the life span of bacteria is relatively short, enabling continuous and natural bacterial turnover, thereby releasing bacterial cell debris into the GIT [28]. The peptidoglycans (PGNs) from the bacterial cell walls are then released and recovered [6]. However, it is still unclear what occurs with the remaining PGN. As birds age, their GIT may accumulate bacterial cell debris, including PGNs; thus, we can assume that the availability and accumulation of PGNs in the birds’ gut at older ages improve the growth performance when using muramidases in the broilers’ feed. Another supporting study concluded that MUR was capable of affecting and degrading PGN in the intestine and increasing the abundance of beneficial bacteria [29].

Muramidase, a member of the lysozyme family, can be considered a new category of feed enzyme. This enzyme will not target substrates from feed grains, but it will hydrolyze PGNs available from broken bacterial cell walls [30,31]. PGN hydrolyzation downregulates the immune response to dead bacterial cell wall debris and thus ensures there are more nutrients and energy available to the bird for growth. One study states that the immune system recognizes PGNs through toll-like receptors (TLRs) and responds with an inflammatory reaction to fight the bacteria [32]. When PGNs are hydrolyzed, using muramidase as in our case, the end product is muramyl dipeptide (MDP); MDP is recognized by NOD-like receptors (NOD2), and when this NOD2 receptor is activated, it actually downregulates an immune response.

The addition of MUR at 350 g/ton and 450 g/ton improved the BW by 86 g and 136 g, respectively, compared to the control diet. Other studies highlighted that higher growth performance shows higher carotenoid concentration in the blood [10,25,33]. This factor is related to higher nutrient absorption and consequently determines the growth performance of the broiler. The enhancement of the BW, DWG, and DFI while adding MUR to the broiler feed was mainly due to the better absorption and nutrient digestibility. In this study, the blood carotenoid concentration was elevated with the highest dose of MUR. This higher blood concentration of total carotenoids indicates a higher health status and a more uniform poultry product when MUR is applied to poultry diets, due to improved growth performance and absorption capacity [6,27].

The low carotenoid levels shown in T2 and T1 could be related to local inflammation or oxidative stress. This factor is not considered in our study because the broilers were healthy throughout the 42-day study duration. Using carotenoids as a biomarker in this study, we evaluated the availability and effectiveness of Apo-ester and showed the enhanced functionality of the broilers’ GIT. The same results were obtained using carotenoids as biomarkers for nutrient absorption and intestinal health [34], and similar results showed an increase in total blood carotenoids when MUR was used in broiler feeds [33]. This enhanced growth performance in 42-day-old broilers was due to improved digestibility. Other studies have demonstrated that MUR is a feed enzyme that does not act on the feed itself; instead, it acts on the PGNs that mitigate intestinal inflammation, which produces muramyl-dipeptide [33]. However, when bird guts are inflamed, the gastrointestinal tract exhibits a decreased digestive efficiency and reduced absorption of both macro- and micronutrients, leading to inadequate nutrition, and in our case, lower broiler performance [35,36].

Carotenoid absorption in the birds’ gut is altered by different factors that may increase or decrease the absorption, such as the shed environment, birds’ feed, or disease [37]. A damaged gut may affect the metabolism and absorption of certain nutrients, and based on our study, a damaged gut will affect the availability of carotenoids and lower growth performance. Furthermore, the use of a fermentable fiber-based diet and coccidiosis vaccine has been highlighted to reduce total carotenoid concentration in the blood [38]. These factors interfere with nutrient absorption and are, consequently, detrimental to birds’ growth performance. In addition, several bird species become pale in captivity, irrespective of carotenoid supplementation, indicating that environmental conditions could be among the major factors regulating carotenoid metabolism in birds [39]. Low carotenoid concentrations may therefore be associated with the low number of enterocytes and length of the villus for absorbing nutrients, as shown in our study using feed without MUR [40]. The digestion and absorption of carotenoids is routinely assessed in research facilities, but much of the technology and equipment used for this purpose is not available for near-commercial farms. In this study, using an iCheck Carotene device, blood samples were analyzed to determine the total carotenoid levels. This process was completed at the farm and took 5 min and 10 s.

MUR is unique in that it functions as an enzyme to hydrolyze the PGNs of dead bacteria. Research has shown that MUR enhances nutrient absorption [30,31], which our results support based on the higher growth performance and higher carotenoid levels observed while using MUR in the broiler feeds. The addition of muramidase in broiler feeds could therefore effectively degrade PGNs in jejunum and excreta, as well as improve intestinal morphology (IM) and increase the abundance of Lactobacillus and short-chain fatty acid production [19,38]. In this study, we found that adding muramidase to the feed of the broilers showed no significant increase in the goblet cell density in the duodenum, ileum, or jejunum. These results agree with those of another study [10] that observed no effects on the numbers of goblet cells in the ileum of broilers fed the same amounts of muramidase or higher. The higher mean value of goblet cells in T1 may indicate an upcoming disease challenge that was not observed at the time of the trial; this may also be due to goblet cells, which are considered epithelial cells, secreting mucus containing anti-microbial proteins for barrier maintenance purposes [41].

The observed increases in villus height and width across multiple intestinal segments in the MUR-supplemented groups are consistent with an enhanced mucosal absorptive capacity, as a greater villous surface area directly supports more efficient nutrient uptake. Segment-specific and inconsistent changes in crypt depth are biologically plausible and have been reported previously in enzyme supplementation trials [9]. The crypt depth reflects the rate of enterocyte proliferation and mucosal renewal; in the jejunum, T2 birds showed significantly reduced crypt depth (141.60 µm) compared to T1 birds (176.50 µm; *p* = 0.0017), which, alongside an increased villus height, suggests a more mature and efficient epithelial architecture rather than heightened cellular turnover. In contrast, the ileal crypt depth increased modestly in T3 birds (128 µm) compared to T1 birds (105.30 µm; *p* = 0.0145), potentially reflecting a compensatory mucosal response to increased nutrient flux in this segment.

However, using muramidase, other studies have found a significant increase in goblet cell density in the duodenum and jejunum [5,25]. We observed no differences in goblet cells among experimental groups, but did observe higher values in the ileum of the control group (*p* < 0.05); this is likely due to indirect effects of the muramidase, as PGN degradation reduces the inflammatory response, which may reduce goblet cell density and lead to a more beneficial microbiota composition and function. These findings have also been reflected in other studies [24,38].

The beneficial effects of muramidase on broiler growth and higher BW are associated with the results from our study on the GIT, showing a longer villus length and width. These results indicate that using MUR in broiler feeds increased the villus length in the duodenum, ileum, and jejunum. The longer villi increased the nutrient absorption and digestive capacity of the GIT. Our results are also compatible with those of a recent study on adding muramidases to feed to improve the intestinal villi and ileum of broilers [37].

## 5. Conclusions

The supplementation of muramidases at different inclusion rates showed a significant increase in the growth performance of broilers raised under commercial conditions. The beneficial effects were observed in both treatments with 350 g/ton and 450 g/ton of MUR, respectively, where the highest response was seen with the highest dose. We can associate the improved growth performance with the enhanced digestion and absorption capacity of the birds’ digestive tract. In addition, the increased villi length and width with the higher MUR dose improved the birds’ growth performance and increased blood carotenoid levels. Notably, the positive effects of MUR on growth performance were evident in this trial; however, no positive impact on FCR. Further investigations are required to confirm the effects of MUR on FCR and the reduction in goblet cells in the GIT. These investigations should consider birds’ responses to the enzyme through ileal digestibility assays, intestinal morphometry, and inflammatory biomarker measurement to provide a more mechanistic understanding of these findings.

## Figures and Tables

**Table 1:** Ingredients and nutritional composition of the starter, grower, and finisher diets using muramidase.

Ingredients (%)	Starter (1–14 Days)	Grower (15–28 Days)	Finisher (29–42 Days)
Yellow Corn	55.4	57.5	57.7
Soy Bean Meal 47%	36.8	35.9	35.5
Soy Oil	3.1	2.7	3.1
Lime Stone ground	1.3	1.1	1.1
Mono Calcium Phosphate	1	0.5	0.3
dl-Methionine 99%	0.4	0.38	0.36
Sodium Bicarbonate	0.3	0.32	0.42
l-Lysine 95%	0.37	0.27	0.3
l-Threonine	0.2	0.17	0.19
Toxin Binder	0.2	0.2	0.2
Broiler Premix ^123^	0.4	0.4	0.4
l-Valine	0.12	0.19	0.1
Choline Chloride 60%	0.11	0.11	0.11
Enzyme ^4^	0.1	0.1	0.1
Salt	0.2	0.16	0.12
Total	100	100	100
Calculated analysis			
AMEn, Kcal/kg	3022	3050	3145
Crude Protein %	23	21.5	19.5
Calcium %	0.96	0.75	0.72
Av. P %	0.54	0.42	0.36
Dig. Lys %	1.32	1.18	1.08
Dig. Met %	0.76	0.51	0.6
Dig. Thr %	0.92	0.79	0.76
Dig. Trp %	0.22	0.21	0.19
Dig. Arg	1.43	1.26	1.18

Broiler Premix ^123^, raw material composition: Vitamin A 10,000 IU; D3 3333 IU; Vitamin E 50 mg; K3 2.5 mg; B1 2.5 mg; Vitamin B2 7.5 mg; B6 5 mg; B12 25 mg; Ca-pantothenate 15 mg; Niacin 50 mg; Biotin 0.25 mg; Folic acid 1.5 mg; BHT 3.12 mg; Na selenite 300 mg; Calcium iodate 300 mg; Iron-(II)-sulfate monohydrate 80 mg; Copper (II) sulfate pentahydrate 8 mg; Manganese 100 mg; Zinc 80 mg; sulfate monohydrate 40 mg. Carotenoids Apo-ester 40 g (DSM Nutritional Product, Kaiseraugust, Switzerland). Balancius^®^ Product code: 5016277 (DSM Nutritional Product, Kaiseraugust, Switzerland). ^4^ Enzymes: Phytase Ronozyme HiPhos, Ronozyme WX, (DSM Nutritional Product, Kaiseraugust, Switzerland).

**Table 2:** Growth performance of broilers receiving diets containing different amounts of muramidase.

Parameter	Day	Amount of Muramidase in Diet						*p* Value
		T1: 0 g/t		T2: 350 g/t		T3: 450 g/t		
		Mean	Sd	Mean	Sd	Mean	Sd	
Body Weight (g/bird) (*n* = 112)	1	44.21	2.25	44.15	2.24	44.22	2.95	0.9756
	21	901.60 ^a^	80.04	934.00 ^b^	92.39	909.70 ^ab^	87.29	0.0152
	42	2377 ^a^	247.90	2463 ^b^	306.40	2513 ^b^	214.5	0.0005
Daily Weight Gain (g/bird) (*n* = 112)	1–21	40.83 ^a^	3.71	42.37 ^b^	4.30	41.21 ^ab^	4.03	0.0119
	22–42	70.25 ^a^	8.07	72.79 ^a^	10.38	76.33 ^b^	6.11	<0.0001
	1–42	55.54 ^a^	5.85	57.58 ^b^	7.24	58.77 ^b^	5.04	0.0004
Daily Feed Intake (g/bird) (*n* = 8)	1–21	58.99 ^a^	0.76	56.89 ^b^	0.33	57.56 ^c^	0.16	<0.0001
	22–42	146.74 ^a^	0.34	148.62 ^b^	1.00	150.65 ^c^	0.98	<0.0001
	1–42	97.72 ^a^	0.50	97.62 ^a^	0.60	98.90 ^b^	0.12	<0.0001
Feed Conversion Ratio (*n* = 8)	1–21	1.46	0.14	1.35	0.14	1.41	0.14	0.3622
	22–42	2.11	0.24	2.08	0.30	1.99	0.16	0.5596
	1–42	1.78	0.19	1.72	0.22	1.69	0.15	0.6803

^a^, ^b^, ^c^ Means within a row with different superscripts significantly differ at *p* < 0.05. Tukey’s test was applied to compare means; Sd = standard deviation.

**Table 3:** Intestinal morphology of broilers receiving diets containing different amounts of muramidase.

Intestinal Morphology		Amount of Muramidase in Diet						*p* Value
		T1: 0 g/t		T2: 350 g/t		T3: 450 g/t		
		Mean	Sd	Mean	Sd	Mean	Sd	
Duodenum	Villus height (μm) (*n* = 90)	849.30 ^a^	220.70	773.10 ^a^	285.50	1230 ^b^	302.2	<0.0001
	Crypt depth (μm) (*n* = 90)	159.40	80.73	151.20	65.30	156.50	59.81	0.7228
	Villus height/crypt depth ratio (*n* = 90)	7.03	4.46	6.19	3.61	8.88	3.58	0.5215
	Villus width (μm) (*n* = 90)	123.90 ^a^	46.11	106.30 ^b^	31.55	138.20 ^c^	40.14	<0.0001
	Goblet cell density (cells/500 μm) (*n* = 90)	34.33	12.44	23.00	9.04	20.56	14.04	0.0515
Jejunum	Villus height (μm) (*n* = 90)	766 ^a^	276.20	872.60 ^b^	207.10	941.50 ^b^	187.20	<0.0001
	Crypt depth (μm) (*n* = 90)	176.50 ^a^	73.58	141.60 ^b^	63.19	171.40 ^a^	72.21	0.0017
	Villus height/crypt depth ratio (*n* = 90)	5.14 ^a^	3.05	7.38 ^b^	3.66	6.41 ^b^	2.81	<0.0001
	Villus width (μm) (*n* = 90)	96.79 ^a^	38.89	113.20 ^b^	34.51	132.60 ^c^	44.75	<0.0001
	Goblet cell density (cells/500 μm) (n = 90)	25.56	18.64	18.89	9.65	29.78	14.6	0.3059
Ileum	Villus height (μm) (*n* = 90)	511 ^a^	161.90	680.60 ^b^	162.40	591.30 ^c^	188.70	<0.0001
	Crypt depth (μm) (*n* = 90)	105.30 ^a^	38.47	120.80 ^ab^	63.14	128 ^b^	54.51	0.0145
	Villus height/crypt depth ratio (*n* = 90)	5.60 ^a^	2.81	7.12 ^b^	3.21	5.35 ^a^	2.63	<0.0001
	Villus width (μm) (*n* = 90)	103.80 ^a^	29.46	92.72 ^b^	24.53	109.70 ^a^	34.48	0.0007
	Goblet cell density (cells/500 μm) (*n* = 90)	53.67 ^a^	15.88	28.89 ^b^	10.96	37.56 ^ab^	14.07	0.003

^a^, ^b^, ^c^ Means within a row with different superscripts significantly differ at *p* < 0.05. Tukey’s test was applied to compare means; Sd = standard deviation.

**Table 4:** Total carotenoids in blood of broilers receiving diets containing different amounts of muramidase.

	Amount of Muramidase in Diet						*p* Value
	0 g/t		350 g/t		450 g/t		
	Mean	Sd	Mean	Sd	Mean	Sd	
Total carotenoids in blood (mg/L) (*n* = 8)	1.02 ^a^	0.21	1.08 ^a^	0.31	1.75 ^b^	0.67	0.0026

^a^, ^b^ Means within a row with different superscripts significantly differ at *p* < 0.05; Tukey’s test was applied to compare means; Sd = standard deviation.

## Data Availability

The data are available upon reasonable request from the corresponding author (A.K.).

## References

[1] Allen P.C., Fetterer R.H. (2002). Interaction of dietary vitamin E with Eimeria maxima infections in chickens. Poult. Sci..

[2] Amer S.A., Farahat M., Gouda A., Abdel-Wareth A.A.A., Abdel-Warith A.-W.A., Younis E.M., Elshopakey G.E., Baher W.M., Saleh G.K., Davies S.J. (2023). New insights into the effects of microbial muramidase addition in the diets of broiler chickens. Animals.

[3] Apajalahti J.H., Kettunen H., Kettunen A., Holben W.E., Nurminen P.H., Rautonen N., Mutanen M. (2002). Culture-independent microbial community analysis reveals that inulin in the diet primarily affects previously unknown bacteria in the mouse cecum. Appl. Environ. Microbiol..

[4] Gong M., Anderson D., Rathgeber B., MacIsaac J. (2017). The effect of dietary lysozyme with EDTA on growth performance and intestinal microbiota of broiler chickens in each period of the growth cycle. J. Appl. Poult. Res..

[5] Adeola O., Cowieson A.J. (2011). BOARD-INVITED REVIEW: Opportunities and challenges in using exogenous enzymes to improve nonruminant animal production. J. Anim. Sci..

[6] Bortoluzzi C., Perez-Calvo E., Olsen P.B., van der Vaart S., van Eerden E., Schmeisser J., Eising I., Segobola P., Sorbara J.-O.B. (2023). Effect of microbial muramidase supplementation in diets formulated with different fiber profiles for broiler chickens raised under various coccidiosis management programs. Poult. Sci..

[7] Brugaletta G., De Cesare A., Laghi L., Manfreda G., Zampiga M., Oliveri C., Pérez-Calvo E., Litta G., Lolli S., Sirri F. (2022). A multi-omics approach to elucidate the mechanisms of action of a dietary muramidase administered to broiler chickens. Sci. Rep..

[8] Callewaert L., Michiels C.W. (2010). Lysozymes in the animal kingdom. J. Biosci..

[9] Cho S., Kumar S.S., Ramirez S., Valientes R., Kim I.H. (2024). Dietary eubiotics of microbial muramidase and glycan improve intestinal villi, ileum microbiota composition and production trait of broiler. J. Anim. Sci. Biotechnol..

[10] Rychen G., Aquilina G., Azimonti G., Bampidis V., Bastos M.D.L., Bories G., Chesson A., Flachowsky G., Gropp J., EFSA Panel on Additives and Products or Substances used in Animal Feed (FEEDAP) (2018). Safety and efficacy of muramidase from Trichoderma reesei DSM 32338 as a feed additive for chickens for fattening and minor poultry species. EFSA J..

[11] EL-Deep M.H., Amber K.A., Eid Y.Z., Alrashood S.T., Khan H.A., Sakr M.S., Dawood M.A.O. (2020). The influence of dietary chicken egg lysozyme on the growth performance, blood health, and resistance against Escherichia coli in the growing rabbits’ cecum. Front. Vet. Sci..

[12] Fleming A. (1922). On a remarkable bacteriolytic element found in tissues and secretions. Proc. R. Soc. Lond. B Biol. Sci..

[13] Fuller R. (1978). Epithelial attachment and other factors controlling the colonization of the intestine of the gnotobiotic chicken by lactobacilli. J. Appl. Bacteriol..

[14] Goes E.C., Dal Pont G.C., Maiorka A., Bittencourt L.C., Bortoluzzi C., Fascina V.B., Lopez-Ulibarri R., Perez Calvo E., Beirão B.C.B., Caron L.F. (2022). Effects of a microbial muramidase on the growth performance, intestinal permeability, nutrient digestibility, and welfare of broiler chickens. Poult. Sci..

[15] Goodarzi Boroojeni F., Männer K., Rieger J., Calvo E.P., Zentek J. (2019). Evaluation of a microbial muramidase supplementation on growth performance, apparent ileal digestibility, and intestinal histology of broiler chickens. Poult. Sci..

[16] Hedl M., Li J., Cho J.H., Abraham C. (2007). Chronic stimulation of Nod2 mediates tolerance to bacterial products. Proc. Natl. Acad. Sci. USA.

[17] Hudon J. (1994). Biotechnological applications of research on animal pigmentation. Biotechnol. Adv..

[18] Humann J., Lenz L.L. (2009). Bacterial peptidoglycan degrading enzymes and their impact on host muropeptide detection. J. Innate. Immun..

[19] Knoop K.A., Newberry R.D. (2018). Goblet cells: Multifaceted players in immunity at mucosal surfaces. Mucosal Immunol..

[20] Lee W.J., Hase K. (2014). Gut microbiota–generated metabolites in animal health and disease. Nat. Chem. Biol..

[21] Lichtenberg J., Perez Calvo E., Madsen K., Lund T.Ø., Birkved F.K., van Cauwenberghe S., Mourier M., Wulf-Andersen L., Jansman A.J.M., Lopez-Ulibarri R. (2017). Safety evaluation of a novel muramidase for feed application. Regul. Toxicol. Pharmacol..

[22] May K.D., Wells J.E., Maxwell C.V., Oliver W.T. (2012). Granulated lysozyme as an alternative to antibiotics improves growth performance and small intestinal morphology of 10-day-old pigs. J. Anim. Sci..

[23] Mayer C. (2012). Bacterial Cell Wall Recycling. Encyclopedia of Life Sciences.

[24] Pérez-Calvo E., Aureli R., Sorbara J.O.B., Cowieson A.J. (2023). Dietary muramidase increases ileal amino acid digestibility of wheat and corn-based broiler diets without affecting endogenous amino acid losses. Poult. Sci..

[25] Pirgozliev V.R., Mansbridge S.C., Westbrook C.A., Woods S.L., Rose S.P., Whiting I.M., Yovchev D.G., Atanasov A.G., Kljak K., Staykova G.P. (2020). Feeding dihydroquercetin and vitamin E to broiler chickens reared at standard and high ambient temperatures. Arch. Anim. Nutr..

[26] Reith J., Mayer C. (2011). Peptidoglycan turnover and recycling in gram-positive bacteria. Appl. Microbiol. Biotechnol..

[27] Rochell S.J., Parsons C.M., Dilger R.N. (2016). Effects of Eimeria acervulina infection severity on growth performance, apparent ileal amino acid digestibility, and plasma concentrations of amino acids, carotenoids, and α1-acid glycoprotein in broilers. Poult. Sci..

[28] Sais M., Barroeta A.C., López-Colom P., Nofrarías M., Majó N., Lopez-Ulibarri R., Calvo E.P., Martín-Orúe S.M. (2020). Evaluation of dietary supplementation of a novel microbial muramidase on gastrointestinal functionality and growth performance in broiler chickens. Poult. Sci..

[29] Ceylan N., Bortoluzzi C., Gunturkun O., Perez-Calvo E. (2024). Comparative effects of dietary muramidase and phytogenics on the growth performance and gastrointestinal functionality of broiler chickens. Poult. Sci..

[30] Stevens L. (1991). Egg white proteins. Comp. Biochem. Physiol. B.

[31] Traub S., von Aulock S., Hartung T., Hermann C. (2006). Invited review: MDP and other muropeptides—Direct and synergistic effects on the immune system. J. Endotoxin Res..

[32] Vidal M.L., Gautron J., Nys Y. (2005). Development of an ELISA for quantifying lysozyme in hen egg white. J. Agric. Food. Chem..

[33] Walk C.L., Alleno C., Bouvet R., Thoby J.-M., Eising I., Segobola P. (2023). Dietary muramidase improved growth performance, feed efficiency, breast meat yield, and welfare of turkeys from hatch to market. Poult. Sci..

[34] Vollmer W., Seligman S.J. (2010). Architecture of peptidoglycan: More data and more models. Trends Microbiol..

[35] Celi P., Verlhac V., Pérez Calvo E., Schmeisser J., Kluenter A.M. (2019). Biomarkers of gastrointestinal functionality in animal nutrition and health. Anim. Feed. Sci. Technol..

[36] Davin R., Manzanilla E.G., Klasing K.C., Pérez J.F. (2012). Evolution of zinc, iron, and copper concentrations along the gastrointestinal tract of piglets weaned with or without in-feed high doses of zinc oxide compared to unweaned littermates. J. Anim. Sci..

[37] Wang Y., Goossens E., Eeckhaut V., Calvo E.P., Lopez-Ulibarri R., Eising I., Klausen M., Debunne N., De Spiegeleer B., Ducatelle R. (2021). Dietary muramidase degrades bacterial peptidoglycan to NOD-activating muramyl dipeptides and reduces duodenal inflammation in broiler chickens. Br. J. Nutr..

[38] Wellman-Labadie O., Picman J., Hincke M.T. (2007). Avian antimicrobial proteins: Structure, distribution and activity. Worlds Poult. Sci. J..

[39] Wheeler R., Chevalier G., Eberl G., Gomperts Boneca I. (2014). The biology of bacterial peptidoglycans and their impact on host immunity and physiology: Bacterial peptidoglycans in the mammalian host. Cell Microbiol..

[40] Xu S., Shi J., Shi X., Dong Y., Wu X., Li Z., Fang Z., Lin Y., Che L., Li J. (2018). Effects of dietary supplementation with lysozyme during late gestation and lactation stage on the performance of sows and their offspring. J. Anim. Sci..

[41] Yegani M., Korver D.R. (2008). Factors affecting intestinal health in poultry. Poult. Sci..

